# 2069: Competency indices for clinical research professionals

**DOI:** 10.1017/cts.2017.158

**Published:** 2018-05-10

**Authors:** Carlton Hornung, Carolyn Thomas Jones, Terri Hinkley, Vicki Ellingrod, Nancy Calvin-Naylor

## Abstract

OBJECTIVES/SPECIFIC AIMS: Clinical research in the 21st century will require a well-trained workforce to insure that research protocols yield valid and reliable results. Several organizations have developed lists of core competencies for clinical trial coordinators, administrators, monitors, data management/informaticians, regulatory affairs personnel, and others. While the Clinical Research Appraisal Inventory assesses the self-confidence of physician scientists to be clinical investigators, no such index exists to assess the competence of clinical research professionals who coordinate, monitor, and administer clinical trials. We developed the Competency Index for Clinical Research Professionals (CICRP) as a general index of competency (ie, GCPs) as well as sub-scales to assess competency in the specific domains of Medicines Development; Ethics and Participant Safety; Data Management; and Research Methods. METHODS/STUDY POPULATION: We analyzed data collected by the Joint Task Force on the Harmonization of Core Competencies from a survey of research professionals working in the United States and Canada. Respondents reported how competent they believed themselves to be on 51 clinical research core competencies. Factor analyzes identified 20 core competencies that defined a Competency Index for Clinical Research Professionals—General (CICRP-General, ie, GCPs) and 4 subindices that define specialized research functions: Medicines Development; Ethics and Participant Safety; Data Management; and Research Concepts. RESULTS/ANTICIPATED RESULTS: Factor analysis identified 20 core competencies that defined a Competency Index for Clinical Research Professionals—General (CICRP-General, ie, GCPs) and 4 subindices that define specialized research functions: Medicines Development; Ethics and Participant Safety; Data Management; and Research Concepts. DISCUSSION/SIGNIFICANCE OF IMPACT: These indices can be used to gage an individual’s readiness to perform general as well as more advanced research functions; to assess the education and training needs of research workers; and to evaluate the impact of education and training programs on the competency of research coordinators, monitors, and other clinical research team members.

